# Polycomb-Focused Transcriptome-Wide Association Study (TWAS) Reveals a Neuronal Identity, Circuit-Maintenance, and Cellular-Logistics Architecture Linking Anorexia Nervosa and Binge-Eating Behaviour

**DOI:** 10.7759/cureus.113917

**Published:** 2026-08-04

**Authors:** Ngo Cheung, Hoi Ki Cheung, Yee-Wah Yu, Tsz-Tin Wu, Yolanda Yuen-Ching Tsang

**Affiliations:** 1 Psychiatry, Cheung Ngo Medical Limited, Hong Kong, HKG; 2 Psychiatry, Chinese University of Hong Kong, Hong Kong, HKG; 3 Psychiatry, University of Hong Kong, Hong Kong, HKG

**Keywords:** aging, anorexia nervosa, binge eating, binge-eating behaviour, h3k27me3, nmn, polycomb, s-predixcan, transcriptome-wide association study

## Abstract

Background

Anorexia nervosa and binge-eating behaviour are clinically distinct yet genetically and neurobiologically overlapping phenotypes. The molecular systems that differentiate restrictive from binge-related eating pathology remain incompletely defined, particularly when genetic risk is integrated with age-related neuronal chromatin programs. A companion preprint prioritized synaptic vesicle dynamics, Ras homolog family member A (RHOA)-centered trafficking, and mitochondrial substrate utilization as candidate interfaces between eating-disorder genetic risk and nicotinamide mononucleotide (NMN)-sensitive biology.

Objective

This study aimed to extend the companion NMN-sensitive framework by applying a Polycomb- and Kyoto Encyclopedia of Genes and Genomes (KEGG)-derived multi-gene-set transcriptome-wide association study (TWAS) to the same brain-tissue summary-statistics-based PrediXcan (S-PrediXcan) results for broad anorexia nervosa (AN broad) and broad binge-eating behaviour (BE broad). The analysis is hypothesis-generating and is not an independent replication of the companion study.

Methods

Sixteen Polycomb- and KEGG-derived gene sets, constructed from age-associated H3K27me3 programs in neurons, were interrogated. Analytical steps included enrichment testing, competitive permutation analysis, bootstrap confidence intervals, disease-differential testing, tissue-level analyses, profile proximity, concordance testing, leave-one-out influence analysis, pooled Benjamini-Hochberg false discovery rate (BH-FDR) correction across the prespecified family of enrichment, differential, proximity, and pairwise tests, network co-occurrence, module clustering, cross-module candidate prioritization, and robustness-weighted prioritization.

Results

The synaptic vesicle cycle showed the strongest positive enrichment in BE broad (Stouffer Z = 4.628; competitive permutation p = 0.0179; Wilcoxon p = 0.00229; bootstrap 95% confidence interval (CI): 0.93-8.23). Positive tissue-level statistics were observed in the frontal cortex BA9, anterior cingulate cortex BA24, and caudate basal ganglia, although these tissues are correlated and were not treated as independent replications. AN broad exhibited its strongest meta-level signal in axon guidance, with additional enrichment involving adherens junctions, long-term potentiation, and ribonucleic acid (RNA) polymerase-related genes.

Stage 3 integration identified four higher-order module clusters, 28 cross-module prioritized candidates influential across at least three modules and both phenotypes, 577 influential candidate genes, and 576 cross-module network nodes. RHOA emerged as a recurrent, high-influence, and directionally unstable network node and candidate linking adhesion, actin remodeling, trafficking, and signaling. Across pathways, the two phenotypes shared substantial molecular infrastructure yet demonstrated only moderate directional concordance, consistent with a dimensional rather than strictly categorical model.

Conclusions

The results supply structural and regulatory context for the companion NMN framework and support a dimensional model in which the two phenotypes share substantial molecular infrastructure yet differ in pathway magnitude and regional weighting. All pathway and gene prioritizations require independent replication, colocalization, fine-mapping, and functional validation before any mechanistic or therapeutic inference can be drawn.

## Introduction

Clinical and genetic heterogeneity of anorexia nervosa and binge-eating behaviour

Anorexia nervosa and binge-eating behaviour are usually discussed as contrasting clinical phenotypes. Anorexia nervosa is associated with restrictive eating, low body weight, fear of weight gain, and persistent disturbance in the valuation of food and body-related signals. Binge-eating behaviour involves episodes of excessive food intake accompanied by impaired control and is commonly linked to altered reward processing, reinforcement learning, and impulse regulation. Although these presentations differ clinically, they can overlap within individuals and across the course of illness. Binge-restrict cycles, diagnostic crossover, metabolic changes, and shared psychiatric comorbidity complicate any model that treats diagnostic categories as biologically homogeneous [1-5].

Recent genetic studies have reinforced this complexity. The large eating-disorder genome-wide association study (GWAS) program that included broad binge-eating behaviour and anorexia nervosa identified both shared and phenotype-specific genetic components. Binge-eating behaviour showed genetic relationships with obesity-related and impulse-control traits, whereas anorexia nervosa showed a contrasting metabolic and anthropometric profile. The two phenotypes also differed in the direction and magnitude of their genetic correlations with metabolic traits, despite sharing psychiatric genetic correlations. These findings support the view that shared genetic liability does not imply identical molecular pathophysiology [1,2]. The distinction is important for the interpretation of downstream molecular studies: the same biological system may be involved in both phenotypes while contributing differently to each.

The neurocircuitry literature is consistent with this interpretation. Anorexia nervosa has been linked to altered reward valuation, cognitive control, interoceptive processing, and taste-reward circuitry, while binge-related phenotypes have been associated with food-cue reactivity, reward anticipation, habitual responding, and frontostriatal function [3-5]. These systems are not isolated. They depend on distributed neuronal networks whose function requires stable circuit architecture, synaptic adhesion, receptor trafficking, presynaptic release, and sufficient metabolic reserve. A genetic analysis that focuses only on individual genes or one pathway may therefore miss the relationship between neuronal structure and synaptic function.

This creates a translational challenge. A pathway may be associated with both anorexia nervosa and binge-eating behaviour, yet its functional effect may differ by region, cell type, developmental history, metabolic context, or degree of neuronal activity. Conversely, a pathway may show a strong aggregate signal in one phenotype while still sharing a substantial gene-level architecture with the other. The aim of the present analysis was not to replace categorical diagnoses but to identify molecular dimensions that may cut across them [1,2,6,7].

Metabolic and aging biology in the nicotinamide mononucleotide (NMN) framework

The companion preprint proposed that NMN-sensitive biology may intersect with eating-disorder genetic risk through three related processes: intracellular trafficking, mitochondrial substrate utilization, and stress-responsive signaling. In that framework, Ras-related protein Rab-11A (RAB11A) represented endosomal recycling, carnitine palmitoyltransferase 2 (CPT2) represented long-chain fatty-acid oxidation, and mitogen-activated protein kinase kinase 2 (MAP2K2) represented a signaling component responsive to metabolic and cellular stress [6,7]. The central premise was that nicotinamide adenine dinucleotide (NAD+) repletion may influence tissue-specific cellular resilience rather than produce one uniform transcriptional response.

This model is biologically plausible because neuronal activity places simultaneous demands on membrane trafficking, ion gradients, neurotransmitter release, and adenosine triphosphate (ATP) production. Synaptic vesicles must be retrieved, refilled, transported, and returned to release sites. Receptor availability depends on endosomal sorting and recycling. Mitochondria must support local energy demand while maintaining redox balance. These processes can be altered by nutrient deprivation, repeated stimulation, oxidative stress, or changes in neuronal activity [8-11].

The companion transcriptome-wide association study (TWAS) analysis prioritized a strong binge-eating association with the synaptic vesicle cycle and highlighted Ras homolog family member A (RHOA), presynaptic genes, and metabolic candidates as experimental entry points [7]. The present analysis therefore asked whether these signals recur when the same TWAS summary statistics are interrogated with gene sets constructed from a distinct Polycomb-focused rationale while recognizing that recurrence under a different gene-set definition does not constitute independent replication.

Polycomb-mediated repression in aging neurons

The Polycomb gene sets used in this study were derived from a secondary analysis of trimethylated histone H3 lysine 27 (H3K27me3) signal in purified mouse forebrain neurons across young adult, middle-aged, and old age. That analysis identified 1,500 age-associated region-gene assignments. H3K27me3 gain predominated, accounting for 1,383 assignments, or 92% of the interpreted set, whereas 117 assignments, or 8%, showed loss. The strongest gain program involved clustered protocadherin genes, including Pcdhb genes, Pcdhgb4, Pcdhgc3, and Pcdha7. A second gain program involved synaptic maintenance and neurotransmission genes, including Nlgn3, Cask, Dlg4, Dlg3, Syn1, Chrm2, Chrna5, and Chrnb4. The smaller loss program included Med14, Med12, Atp6ap2, and Heph, among other transcriptional, lysosomal, and stress-related genes [12].

The proposed interpretation was not that aging neurons simply lose H3K27me3 everywhere. Instead, repression may be redistributed, with focal gain at neuronal identity and synaptic-maintenance loci and selective loss at regulatory, stress, or homeostatic loci. Clustered protocadherins are particularly relevant because they provide combinatorial cell-surface recognition systems that contribute to dendritic self-avoidance and neuronal individuality. Synaptic adhesion and scaffold genes, including neuroligins, CASK, postsynaptic density (PSD)-family proteins, and synapsin, connect neuronal identity to the stability and organization of mature synapses.

This framing introduces a new layer to the eating-disorder analysis. The relevant question is not only whether eating-disorder genetic risk is associated with presynaptic or metabolic genes. It is also whether risk intersects with programs that determine which synaptic partners are maintained, how neuronal processes are organized, and how circuits preserve functional precision over time. A Polycomb-focused analysis therefore provides a hypothesis-generating structural and regulatory context for the trafficking and synaptic signals identified in the companion preprint.

TWAS as a bridge between GWAS and molecular programs

Summary-statistics-based PrediXcan (S-PrediXcan) and related TWAS methods estimate associations between genetically predicted gene expression and complex traits using GWAS summary statistics. The approach provides a gene-level framework for interpreting polygenic risk and can be applied across many tissues. S-PrediXcan was developed to reproduce PrediXcan-style gene-level associations from summary data and has been used to scan broad tissue panels, in part because the relevant regulatory context is not always apparent in advance [13,14].

The method is useful for prioritization but does not establish that a gene is causally responsible for a trait. Correlated expression predictors, linkage disequilibrium (LD), tissue mismatch, prediction-model uncertainty, horizontal pleiotropy, and multiple causal variants can all influence a TWAS association. Colocalization and fine-mapping methods can help distinguish shared expression quantitative trait locus (eQTL) signals from associations caused by linked but distinct variants [15,16]. The interpretation of a pathway-level TWAS result must therefore remain separate from claims about measured expression, protein abundance, neuronal activity, or treatment response.

The present analysis used TWAS results as an intermediate layer between GWAS and biological interpretation. Because the same underlying S-PrediXcan results were used in the companion and Polycomb-focused analyses, the current study represents a re-analysis of an existing resource rather than an independent TWAS replication. The central focus was not the discovery of new GWAS loci, but the identification of coherent gene-set patterns, shared and divergent disease architectures, tissue-specific effects, and candidate genes for experimental follow-up.

Gap and study aim

The companion NMN-sensitive trafficking preprint identified synaptic vesicle dynamics, RHOA-centered trafficking, and mitochondrial substrate utilization as possible interfaces with anorexia nervosa and binge-eating genetic risk. The Polycomb-focused analysis extends that framework by asking whether these processes are positioned within a larger architecture of neuronal identity, circuit maintenance, adhesion, cytoskeletal remodeling, and stress-dependent resilience.

Because the same S-PrediXcan summary statistics are reused, the present work constitutes a gene-set re-analysis under a Polycomb rationale rather than an independent replication; its purpose is prioritization of candidate architectures for experimental follow-up.

The specific objectives were to determine whether the synaptic vesicle signal would recur in Polycomb-derived gene sets, to assess whether neuronal identity and adhesion programs would add a structural dimension to the NMN model, to identify higher-order relationships among the 16 tested modules, to distinguish recurrent cross-disease candidates from phenotype-specific influential genes, to characterize regional heterogeneity, and to generate a staged experimental framework that separates strong pathway evidence from fragile or highly leveraged signals.

## Materials and methods

GWAS and TWAS inputs

**Figure 1:**
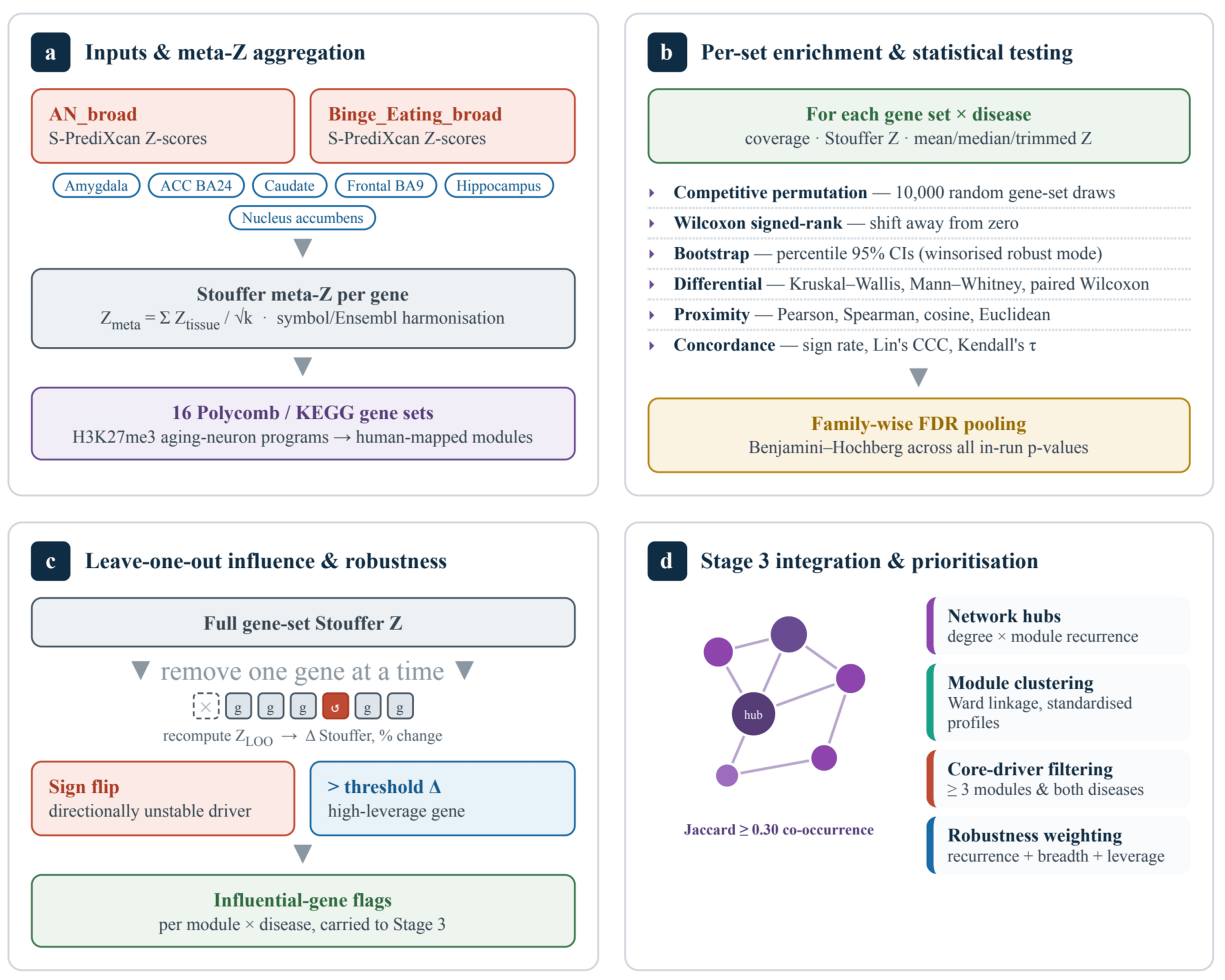
Polycomb-focused multi-gene-set TWAS analytical pipeline (a) Brain-tissue S-PrediXcan Z-scores for both phenotypes across six regions are combined into per-gene Stouffer meta-Z statistics and interrogated with 16 Polycomb- and KEGG-derived gene sets. (b) Each gene set is evaluated per disease using competitive permutation, Wilcoxon, bootstrap confidence intervals, differential, proximity, and concordance testing, with pooled Benjamini-Hochberg FDR correction across the prespecified family of tests. (c) Leave-one-out (jackknife) analysis quantifies each gene's leverage on the aggregate statistic, flagging sign-flipping and high-Δ genes. (d) Stage 3 integrates influential genes into a Jaccard co-occurrence network, clusters modules by Ward linkage, prioritizes cross-module candidates shared across modules and phenotypes, and applies robustness-weighted prioritization. The workflow is hypothesis-generating; no raw TWAS data are re-tested. TWAS: transcriptome-wide association study; KEGG: Kyoto Encyclopedia of Genes and Genomes; FDR: false discovery rate; AN broad: broad anorexia nervosa; CCC: concordance correlation coefficient The image was created by Ngo Cheung using Microsoft PowerPoint (Microsoft Corp., Redmond, WA, USA).

The analysis (Figure 1) used brain-tissue S-PrediXcan results for broad anorexia nervosa (AN broad) and broad binge-eating behaviour (BE broad) derived from the eating-disorder GWAS program and generated using the summary-statistics implementation of PrediXcan [1,13]. These were the same underlying brain-tissue TWAS summary statistics used in the companion analyses, rather than an independent dataset. The disease labels used in the pipeline were AN_broad and Binge_Eating_broad. Tissue-level files were parsed for gene symbols, Ensembl identifiers, and Z-scores. When tissue-specific Z-scores were available for a gene, the pipeline calculated a Stouffer-type meta-Z by summing the tissue Z-scores and dividing by the square root of the number of contributing tissues [17].

Tissue-level Z-scores from the six brain regions are correlated. The simple Stouffer aggregation therefore may overstate precision. We retained the meta-Z primarily for ranking and gave greatest interpretive weight to signals supported by complementary evidence, including competitive permutation, bootstrap confidence intervals excluding zero, positive observations in multiple tissues, disease-differential testing, or pooled Benjamini-Hochberg false discovery rate (BH-FDR) survival. The supplied outputs do not include a tissue covariance matrix or a covariance-adjusted Brown or Kost re-analysis; accordingly, the present manuscript does not claim covariance-adjusted robustness. Tissue-specific results were retained as a sensitivity analysis rather than treated as independent replications.

Computational environment

All data processing, statistical analyses, permutation testing, bootstrap resampling, multiple-testing correction, network analyses, and visualizations were performed in Python (Python Software Foundation, Fredericksburg, Virginia, USA) in a Colab-style environment using Pandas, NumPy, SciPy, scikit-learn, NetworkX, Matplotlib, Seaborn, and related packages. The multi-gene-set run used random seed 42, 10,000 enrichment permutations, 2,000 bootstrap resamples, robust-mode winsorization, bootstrap confidence intervals, pooled BH-FDR correction, a 20% leave-one-out influence threshold, a Jaccard threshold of 0.30, and the supplied automatic clustering procedure, which returned four clusters. Per-tissue enrichment permutations were set to zero in the supplied run, while tissue-level descriptive statistics and Wilcoxon tests were retained. Exact Python and package versions were not preserved in the supplied execution record and are therefore not inferred here. The run generated SHA-256 input manifests for the analysis directories and gene-set inputs.

This aggregation generated meta-level gene statistics as well as tissue-specific gene dictionaries. The analysis included six brain tissues represented in the supplied output: amygdala, anterior cingulate cortex BA24, caudate basal ganglia, frontal cortex BA9, hippocampus, and nucleus accumbens basal ganglia. The tissue-specific results were retained for subsequent heterogeneity analyses rather than treated only as intermediate values.

Gene-set construction and harmonization

The tested collection contained 16 gene sets derived from the Polycomb-focused aging-neuron analysis and its associated Kyoto Encyclopedia of Genes and Genomes (KEGG) mappings [12,18]. The collection included pathways related to neuronal adhesion, axon guidance, adherens junctions, synaptic vesicle cycling, glutamatergic, gamma-aminobutyric acid (GABA)ergic, serotonergic, and cholinergic synapses, long-term potentiation, neuroactive ligand-receptor interaction, lysosome, ferroptosis, regulation of the actin cytoskeleton, ribonucleic acid (RNA) polymerase, spliceosome, and the renin-angiotensin system.

The original Polycomb work used mouse neuronal chromatin data, whereas the TWAS outputs were human gene-level results [12,13]. The supplied pipeline loaded preassembled human-compatible gene matrix transposed (GMT) and TXT gene-set files; the mouse-to-human orthology conversion occurred before the supplied code was executed and was not implemented within the pipeline itself. The supplied code does not identify the upstream orthology database or release, the rule used for one-to-many ortholog relationships, or the exact number of genes excluded during conversion. The downstream pipeline harmonized the resulting gene symbols by exact match followed by Ensembl alias lookup, removed duplicates after aliasing, and reported direct matches, alias-resolved matches, and unmatched genes during execution. Because the upstream mapping table, excluded-gene list, and complete run log were not included in the materials supplied here, the exact number of excluded or unmatched orthologs cannot be reported without reconstructing that upstream step. These files should accompany the final reproducibility deposit [12,19].

Extended multi-gene-set pipeline

For each disease and gene set, the pipeline calculated the number of genes found, coverage, Stouffer Z, mean Z, mean absolute Z, median Z, a trimmed mean, Wilcoxon testing against zero, and a competitive permutation p-value. The permutation procedure sampled genes from the genome-wide TWAS distribution to evaluate whether the observed gene-set statistic was unusually large relative to a random gene set. This is a competitive enrichment test and is not identical to a self-contained test of whether the genes in the set are associated with the phenotype [20].

Bootstrap resampling was used to generate percentile confidence intervals for the Stouffer statistic and related summary measures [21]. The pipeline also calculated disease-differential tests, including paired Wilcoxon testing, Kruskal-Wallis testing, and pairwise Mann-Whitney testing, rank-biserial correlations, and mean Z-score differences [22-24]. Profile proximity was evaluated with Pearson correlation, Spearman correlation, cosine similarity, Euclidean distance, Manhattan distance, and Lin's concordance correlation coefficient (CCC) [25].

Cross-disease concordance was assessed using sign-concordance rates, binomial testing, Lin's CCC, Kendall's tau, and concordance or discordance counts. A single Benjamini-Hochberg adjustment was applied to the prespecified family of p-values generated across enrichment, differential, pairwise, and proximity analyses. In the remainder of the manuscript, this is referred to as pooled BH-FDR across the prespecified test family; it should not be interpreted as family-wise error-rate control [26].

Stage 3 integrative analyses

Stage 3 used the combined enrichment, influence, pairwise, proximity, and gene-detail outputs. The influential gene network was based on a gene-by-gene-set incidence matrix in which a gene was marked as present when it was flagged as influential in a given module. Pairwise gene co-occurrence was quantified by the Jaccard index [27]. Edges were retained at a Jaccard threshold of 0.30. Network degree and recurrence across modules were then used to identify cross-module hubs.

Module clustering used the disease-level Stouffer profiles and the number of influential genes per module. Features were standardized and clustered by Ward linkage [28]. The number of clusters was selected automatically in the supplied Stage 3 code, yielding four clusters.

Cross-module prioritized candidates were defined as genes influential in at least three modules and in both diseases. Phenotype-specific genes were defined as genes influential in one disease but not the other. Robustness-weighted prioritization combined module recurrence, disease breadth, maximum influence, and the presence of sign flips. Because the supplied score assigns positive weight to the presence of a sign flip, it is more accurately interpreted as a composite prioritization index than as a conventional stability score. Sign flips indicate that a gene is influential but directionally unstable.

Statistical thresholds and qualifications

The principal thresholds were raw p < 0.05, FDR < 0.05, absolute Stouffer Z > 1.96, absolute Cohen's d > 0.5, absolute Lin's CCC > 0.5, sign-concordance rate > 0.70, absolute Kendall's tau > 0.30, absolute Pearson correlation > 0.30, and absolute rank-biserial correlation > 0.30. The multi-gene-set run used 10,000 enrichment permutations, bootstrap confidence intervals [21], robust-mode winsorization for selected statistics, and pooled BH-FDR correction across the prespecified test family [26].

The simple Stouffer aggregation assumes a degree of independence that is unlikely to be fully satisfied across correlated brain tissues. Methods such as Brown's and Kost's approaches can incorporate dependence among component statistics [29,30]. A covariance-adjusted re-analysis was not included in the supplied outputs, and no claim is made that the ranking is invariant under such a correction. The analysis was therefore interpreted as hypothesis-generating. Greatest weight was given to findings supported by more than one type of evidence, especially competitive permutation testing, bootstrap intervals, multi-tissue observations, disease-differential testing, and pooled BH-FDR.

## Results

Conceptual convergence on the synaptic vesicle cycle strengthens the BE entry point

The most robust computational prioritization in the Polycomb-focused analysis was positive enrichment of the synaptic vesicle cycle in BE broad. The meta-level Stouffer Z was 4.628, based on 53 genes and 67% coverage. The competitive permutation p-value was 0.0179, the Wilcoxon p-value was 0.00229, and the bootstrap 95% confidence interval (CI) was 0.93-8.23. This remains the only meta-level finding whose bootstrap interval excludes zero and that survives the pooled BH-FDR threshold; it is therefore the most robust prioritization for functional follow-up.

The tissue-level pattern provided multi-region descriptive support. The synaptic vesicle cycle showed a Stouffer Z of 3.241 in the frontal cortex BA9, 2.489 in the anterior cingulate cortex BA24, and 2.141 in the caudate basal ganglia. These observations indicate positive statistics in several tissues but do not represent independent replication because the tissue-level statistics are correlated. At the same time, the result was not uniformly positive across all tissues. The synaptic vesicle cycle in AN broad had a much smaller meta-level signal, with a Stouffer Z of approximately 0.67, and showed tissue variability ranging from negative values in the amygdala to positive values in the frontal cortex.

The pathway also differentiated the two phenotypes. For the paired gene-level comparison, the mean Z difference for AN broad minus BE broad was −0.609. The Mann-Whitney p-value was 0.01069, and the paired Wilcoxon p-value was 0.04042. The rank-biserial correlation was 0.288, indicating a moderate rather than large distributional difference. The synaptic vesicle cycle was also represented among the pooled BH-FDR-significant entries through its enrichment, differential, proximity, and additional pairwise statistics.

The principal meta-level enrichment results are summarized in Table 1.

**Table 1:** Meta-level enrichment across the 16 tested modules Data analysis: tissue-level Z-scores were combined with a Stouffer meta-Z; gene-set evidence was summarized with competitive permutation and Wilcoxon tests, bootstrap confidence intervals, and pooled BH-FDR correction across the prespecified test family. HSA: homo sapiens; AN: anorexia nervosa; BE: binge-eating behaviour; CI: confidence interval; NR: not reported; perm.: permutation

Gene set	AN Z	BE Z	AN − BE	AN perm. p	BE perm. p	Summary classification
HSA04721_SYNAPTIC_VESICLE_CYCLE	+0.666	+4.628	−3.962	0.7446	0.0179	Primary robust BE signal; bootstrap CI (0.93, 8.23)
HSA04360_AXON_GUIDANCE	+3.147	+0.370	+2.777	0.1323	NR	AN-leading; competitive test not significant
HSA04614_RENIN_ANGIOTENSIN_SYSTEM	−2.760	−2.540	−0.220	0.1843	0.1971	Negative signal in both phenotypes
HSA04520_ADHERENS_JUNCTION	−2.678	−1.793	−0.885	0.2044	0.3727	AN-leading negative signal
HSA04720_LONG_TERM_POTENTIATION	+2.569	−0.280	+2.849	0.2231	NR	Opposite-sign disease contrast
HSA03020_RNA_POLYMERASE	−2.506	−0.330	−2.176	0.2288	NR	AN-leading negative signal
HSA04726_SEROTONERGIC_SYNAPSE	+0.410	+2.357	−1.947	NR	0.2391	BE-leading suggestive signal
HSA03040_SPLICEOSOME	−0.060	+2.192	−2.252	NR	0.2776	BE-leading opposite-sign contrast
HSA04724_GLUTAMATERGIC_SYNAPSE	+1.115	−0.580	+1.695	0.5924	NR	Opposite-sign disease contrast
HSA04514_CELL_ADHESION_MOLECULES_CAMS	+0.230	+1.452	−1.222	NR	0.4762	Shared profile with BE-leading magnitude
HSA04142_LYSOSOME	−1.752	+1.763	−3.515	0.4032	0.3806	Opposite-sign disease contrast
HSA04725_CHOLINERGIC_SYNAPSE	−0.090	−1.025	+0.935	NR	0.6084	Negative BE-leading signal
HSA04727_GABAERGIC_SYNAPSE	−1.214	−1.374	+0.160	0.5574	0.4848	Negative signal in both phenotypes
HSA04080_NEUROACTIVE_LIGAND_RECEPTOR_INTERACTION	+1.757	+1.772	−0.015	0.4012	0.3868	Similar aggregate magnitude
HSA04216_FERROPTOSIS	+0.190	+0.700	−0.510	NR	NR	Weak positive BE-leading signal
HSA04810_REGULATION_OF_ACTIN_CYTOSKELETON	−0.010	−0.030	+0.020	NR	NR	Influence-rich but not enriched at the meta-level

The influential genes mapped onto several sequential stages of presynaptic transmission. SLC17A7 encodes vesicular glutamate transporter 1 (VGLUT1), which loads glutamate into synaptic vesicles and can influence quantal size. ATP6V1E2 is part of the vacuolar-type adenosine triphosphatase (vATPase) machinery that establishes the proton electrochemical gradient required for transmitter loading. Vesicle-associated membrane protein 2 (VAMP2), syntaxin-binding protein 1 (STXBP1), complexin-2 (CPLX2), synaptotagmin-1 (SYT1), and related proteins participate in vesicle docking, priming, calcium sensing, and fusion. CACNA1B encodes a neuronal voltage-gated calcium-channel pore-forming subunit, while DNM1 and DNM2 contribute to membrane scission and vesicle retrieval. The functional roles of calcium channels, vesicle fusion machinery, and active-zone organization provide biological context for prioritizing this group as a candidate presynaptic-throughput program rather than a generic neuronal pathway [31-34]. The central role of presynaptic calcium channels in controlling the timing and strength of neurotransmitter release is well established [32].

When the same TWAS summary statistics are interrogated with an independently constructed Polycomb-derived gene-set collection, the synaptic vesicle cycle again emerges as the strongest binge-eating-associated module, supporting conceptual convergence on presynaptic biology. This is not an independent replication, because the underlying GWAS/TWAS resource is shared [7,12].

The pattern is consistent with a hypothesis that binge-eating genetic risk may intersect with several coordinated steps of presynaptic throughput. The signal does not indicate that every presynaptic gene is uniformly upregulated or downregulated in people with binge-eating behaviour. Rather, it indicates that the distribution of genetically predicted expression associations across a functionally connected vesicle system is shifted in aggregate. This distinction prioritizes functional assays of quantal size, release probability, vesicle acidification, recycling kinetics, synaptic fatigue, and recovery after repeated activity.

The connection between vesicle acidification and recycling is also biologically relevant to the NMN framework. Farsi and colleagues showed that clathrin-coated synaptic vesicles contain the vATPase complex but that clathrin coating suppresses ATP-dependent acidification until uncoating occurs. Removing the coat restored acidification, suggesting that vesicle formation and refilling are temporally coordinated processes [35]. In the context of the present results, ATP6V1E2 could represent a candidate link between membrane recycling, proton-gradient formation, transmitter loading, and the energetic cost of repeated synaptic activity.

The significant tissue-specific signals are summarized in Table 2.

**Table 2:** Tissue-specific signals with absolute Stouffer Z greater than 1.96 Data analysis: tissue-specific Stouffer statistics and Wilcoxon tests were calculated within each tissue and phenotype; rows were selected using |Z| > 1.96. These tissue-level results are descriptive and do not represent independent replication across regions. AN: anorexia nervosa; BE: binge-eating behaviour

Gene set	Disease	Tissue	Z	Wilcoxon p
HSA04360_AXON_GUIDANCE	AN broad	Frontal cortex BA9	+3.430	0.003879
HSA04721_SYNAPTIC_VESICLE_CYCLE	BE broad	Frontal cortex BA9	+3.241	0.001623
HSA03040_SPLICEOSOME	BE broad	Nucleus accumbens	+3.202	0.006318
HSA04724_GLUTAMATERGIC_SYNAPSE	BE broad	Amygdala	−2.951	0.01375
HSA04614_RENIN_ANGIOTENSIN_SYSTEM	AN broad	Nucleus accumbens	−2.925	0.1641
HSA04727_GABAERGIC_SYNAPSE	BE broad	Amygdala	−2.552	0.0263
HSA04721_SYNAPTIC_VESICLE_CYCLE	BE broad	Anterior cingulate cortex BA24	+2.489	0.01825
HSA04614_RENIN_ANGIOTENSIN_SYSTEM	BE broad	Nucleus accumbens	−2.393	0.1641
HSA03020_RNA_POLYMERASE	AN broad	Amygdala	−2.387	0.01099
HSA04520_ADHERENS_JUNCTION	BE broad	Caudate	−2.252	0.08842
HSA04721_SYNAPTIC_VESICLE_CYCLE	BE broad	Caudate	+2.141	0.01600
HSA03040_SPLICEOSOME	BE broad	Amygdala	+2.132	0.07949
HSA04360_AXON_GUIDANCE	AN broad	Anterior cingulate cortex BA24	+2.111	0.04385
HSA04614_RENIN_ANGIOTENSIN_SYSTEM	AN broad	Hippocampus	−1.990	0.2500
HSA04520_ADHERENS_JUNCTION	AN broad	Caudate	−1.971	0.6080

A neuronal identity and circuit-precision axis emerges as a major hypothesis-generating contribution

A major hypothesis-generating contribution of the current analysis is the addition of a neuronal identity and circuit-precision axis. The Polycomb-focused gene sets included pathways derived from the protocadherin and synaptic-adhesion modules of the aging-neuron study. In the eating-disorder TWAS results, these programs were represented by cell adhesion molecules, axon guidance, adherens junctions, actin-cytoskeleton regulation, and related synaptic pathways.

Axon guidance was the strongest meta-level signal in AN broad. It had a Stouffer Z of 3.147, although its competitive permutation p-value was 0.1323 and its bootstrap interval extended from −0.17 to 6.80. The strongest tissue-level result was in the frontal cortex BA9, with a Stouffer Z of 3.430. The anterior cingulate cortex BA24 also showed a positive signal, with a Z of 2.111. Although axon guidance produced a large Stouffer Z in AN broad, the competitive permutation test was non-significant (p = 0.132). The signal is therefore regarded as exploratory and is reported primarily for its tissue-level consistency in the frontal cortex BA9. These findings are compatible with, but do not establish, anorexia nervosa-related involvement of neuronal wiring and circuit organization.

Cell adhesion molecules showed the highest cross-disease profile proximity in the analysis. Pearson correlation was 0.549, Spearman correlation was 0.399, and Lin's CCC was 0.547. The cell-level sign-concordance rate was 0.604, and Kendall's tau was 0.281. Thus, the two phenotypes showed moderate-to-strong similarity in the relative ranking of gene-level effects, even though their overall pathway magnitudes and individual directions were not identical.

The corresponding cross-disease proximity and concordance metrics are summarized in Table 3.

**Table 3:** Cross-disease profile proximity and concordance Data analysis: Pearson and Spearman correlations, cosine similarity, Euclidean and Manhattan distances, Lin's CCC, and sign-concordance statistics were calculated from paired gene-level TWAS Z-scores. CCC: concordance correlation coefficient; TWAS: transcriptome-wide association study

Gene set	n	Pearson's r	Spearman's rho	Sign concordance	Lin's CCC
HSA04514_CELL_ADHESION_MOLECULES_CAMS	108	0.549	0.399	0.604	0.547
HSA04520_ADHERENS_JUNCTION	66	0.516	0.380	0.646	0.497
HSA04216_FERROPTOSIS	32	0.434	0.391	0.613	0.433
HSA04726_SEROTONERGIC_SYNAPSE	78	0.394	0.367	0.603	0.390
HSA04724_GLUTAMATERGIC_SYNAPSE	83	0.391	0.422	0.590	0.388
HSA04360_AXON_GUIDANCE	136	0.371	0.312	0.634	0.355
HSA03040_SPLICEOSOME	97	0.371	0.358	0.649	0.366
HSA04721_SYNAPTIC_VESICLE_CYCLE	53	0.323	0.302	0.585	0.308
HSA04810_REGULATION_OF_ACTIN_CYTOSKELETON	167	0.244	0.305	0.632	0.244
HSA04720_LONG_TERM_POTENTIATION	48	0.141	0.340	0.604	0.138

These findings prioritize processes that precede or accompany mature synaptic transmission. Clustered protocadherins provide combinatorial cell-surface recognition systems that contribute to neuronal self-recognition, dendritic self-avoidance, and the organization of local circuits. Experimental work has shown that protocadherins are required for dendritic self-avoidance in mammalian neurons, while more recent work has emphasized the importance of clustered protocadherin cis-interactions for combinatorial neuronal recognition [36,37]. The aging-neuron Polycomb analysis proposed that focal H3K27me3 gain at these loci could compress the molecular diversity that supports neuronal individuality [12].

The current TWAS results do not demonstrate that protocadherin genes are repressed in anorexia nervosa or binge-eating behaviour. They do, however, suggest that the genetic architecture of the two phenotypes intersects with pathways involved in adhesion, axonal pathfinding, synaptic partner selection, and cytoskeletal positioning. This adds a structural dimension to the companion preprint. The resulting hypothesis is not that circuit architecture has been shown to be altered, but that connectivity, adhesion, and receptor organization should be examined alongside presynaptic vesicle function.

Four higher-order module clusters reveal biological coherence

Stage 3 clustering identified four higher-order groups from the 16 tested gene sets. The clusters were generated using standardized disease-level enrichment profiles and influence density, so they should be interpreted as data-driven relationships among the tested modules rather than as definitive biological classifications.

Cluster 1 contained RNA polymerase, adherens junction, GABAergic synapse, and renin-angiotensin system pathways. This group combined transcriptional machinery with adhesion, inhibitory synaptic signaling, and a vascular or neuroendocrine signaling pathway. Its structure is consistent with a set of programs that may reflect regulatory state, cell-cell organization, and neuromodulatory context.

Cluster 2 contained cell adhesion molecules, ferroptosis, lysosome, neuroactive ligand-receptor interaction, serotonergic synapse, and synaptic vesicle cycle. This was the most integrative cluster because it joined presynaptic transmission with membrane adhesion, lysosomal processing, neurotransmitter signaling, and cellular stress. Its composition is consistent with the hypothesis that the strongest binge-eating synaptic vesicle signal is embedded within a broader cellular-maintenance architecture rather than representing an isolated vesicle pathway.

Cluster 3 contained glutamatergic synapse, axon guidance, and long-term potentiation. This grouping was particularly relevant to the proposed circuit architecture-to-synaptic throughput model. It connects the organization of neuronal projections with excitatory transmission and activity-dependent plasticity.

Cluster 4 contained spliceosome, cholinergic synapse, and regulation of the actin cytoskeleton. This group suggests an interface between RNA processing, neuromodulatory signaling, and the actin-dependent organization of membrane and synaptic structures. It also helps explain why actin regulation was highly represented in the influence network despite having little overall meta-level enrichment.

The four higher-order module clusters are summarized in Table 4.

**Table 4:** Stage 3 module clusters Data analysis: standardized disease-level enrichment and influence-density features were clustered using Ward hierarchical linkage; the number of clusters was selected by the supplied Stage 3 procedure.

Cluster	Number of modules	Modules
1	4	HSA03020_RNA_POLYMERASE; HSA04520_ADHERENS_JUNCTION; HSA04727_GABAERGIC_SYNAPSE; HSA04614_RENIN_ANGIOTENSIN_SYSTEM
2	6	HSA04514_CELL_ADHESION_MOLECULES_CAMS; HSA04216_FERROPTOSIS; HSA04142_LYSOSOME; HSA04080_NEUROACTIVE_LIGAND_RECEPTOR_INTERACTION; HSA04726_SEROTONERGIC_SYNAPSE; HSA04721_SYNAPTIC_VESICLE_CYCLE
3	3	HSA04724_GLUTAMATERGIC_SYNAPSE; HSA04360_AXON_GUIDANCE; HSA04720_LONG_TERM_POTENTIATION
4	3	HSA03040_SPLICEOSOME; HSA04725_CHOLINERGIC_SYNAPSE; HSA04810_REGULATION_OF_ACTIN_CYTOSKELETON

The clustering results complement, but do not replace, the original Polycomb Modules A, B, and C. Module A emphasized protocadherin and neuronal adhesion programs. Module B emphasized synaptic scaffold, neurotransmission, and receptor biology. Module C emphasized selective loss at transcriptional, lysosomal, stress, and cytoskeletal loci. The Stage 3 clusters reorganized these concepts according to their behaviour in the human anorexia nervosa and binge-eating TWAS data.

A hypothesis-generating circuit architecture-to-synaptic throughput model

The combined results are consistent with a hypothesis-generating model rather than a demonstrated causal chain. In the proposed model, Polycomb-related neuronal identity and adhesion programs provide a structural layer that may influence circuit specificity, axon guidance, synaptic organization, and receptor positioning. These processes may interact with membrane trafficking and cytoskeletal signaling, which in turn could influence presynaptic vesicle loading, release probability, fusion, and recycling. Any resulting effects on reward processing, reinforcement learning, food-cue reactivity, or cognitive control remain hypotheses requiring direct testing.

This model should be understood as a hypothesis about biological organization rather than a demonstrated causal chain. It is supported by the convergence of pathways, not by direct measurements of chromatin, gene expression, protein abundance, or synaptic physiology in patients.

Several prioritized candidates provide a plausible bridge between the structural and presynaptic layers. RHOA, ITGB1, SRC, PIK3CA, PIK3CB, PTK2, PAK2, PAK4, LIMK2, MYL9, VCL, ARPC5L, and SSH2 connect adhesion receptors, focal-adhesion signaling, phosphoinositide 3-kinase (PI3K) and mitogen-activated protein kinase (MAPK) pathways, actin remodeling, and membrane movement. These genes were recurrently influential in the current data. The network also identified ITGB1, PIK3CA, PIK3CB, PIK3CD, PIK3R2, SRC, MYL9, and RHOA among the highest-degree cross-module hubs.

This model provides a testable explanation for why cell adhesion, axon guidance, adherens junction, actin, and synaptic modules show related patterns. Presynaptic function is not independent of neuronal architecture. Calcium channels must be trafficked to active zones, synaptic vesicles must be transported along cytoskeletal tracks, receptors must be internalized and recycled, and adhesion complexes must stabilize contacts between neurons. Cytoskeletal and membrane-trafficking systems could therefore influence the gain, timing, and persistence of synaptic signaling [38,39].

RHOA is prioritized as a high-influence but context-dependent candidate

RHOA was one of the clearest recurrent candidate signals across the companion NMN-sensitive trafficking preprint and the Polycomb-focused analysis. In the present dataset, RHOA was influential in three modules and both diseases. Its mean absolute percentage influence was approximately 165.8%, its maximum influence was 535.9%, and it had two sign flips in the cross-module prioritization table. Its robustness-prioritization score was approximately 0.850. In the co-occurrence network, RHOA had degree 191 and was among the leading cross-module hubs.

RHOA's biological position makes it a plausible experimental candidate. RHOA and RHO-kinase signaling can regulate actin organization, membrane trafficking, and the movement or retention of membrane proteins. RHOA-related signaling can interact with dynamin, LIM domain kinase (LIMK), myosin, focal-adhesion components, and phosphoinositide pathways. Experimental work has shown that RHOA/RHO-kinase signaling can have dual effects on the trafficking of voltage-sensitive channels, illustrating why its effect may differ across cellular states [40]. Rho guanosine triphosphatases (GTPases) also participate broadly in intracellular transport and cytoskeletal remodeling [41].

The Ras-related protein Rab-5 (RAB5) and Ras-related protein Rab-11 (RAB11) systems provide a complementary trafficking framework. RAB5 is associated with early endosomal organization, whereas RAB11 regulates later recycling through recycling endosomes. Rab activation is controlled by context-dependent exchange and regulatory mechanisms rather than by a simple on-off pathway [42]. This makes RHOA a testable candidate for state-dependent effects on receptor recycling and vesicle movement, rather than evidence of a uniform disease direction.

The sign-flip pattern is central to the interpretation. A sign flip means that removal of RHOA changes the direction of the aggregate Stouffer statistic. This is evidence of leverage and instability, not proof of uniform activation or inhibition. The repeated sign changes may reflect genuine context dependence, differences among brain regions, correlated genes within the pathway, or sensitivity to a relatively extreme gene-level Z-score. RHOA is therefore a high-priority candidate for activity- and metabolic-state-dependent functional assays; network centrality alone does not establish it as a disease mechanism.

Priority assays would measure GTP-bound RHOA (RHOA-GTP), Rho-associated coiled-coil-containing protein kinase (ROCK) and LIMK activity, actin organization, RAB5 and RAB11 compartment dynamics, endosomal pH, receptor internalization, and cargo recycling. These assays should be performed under baseline and stress conditions and should compare anorexia nervosa-relevant and binge-eating-relevant cellular contexts.

Shared molecular infrastructure with phenotype-divergent weighting

The cross-disease analyses showed that AN broad and BE broad share molecular infrastructure but differ in quantitative weighting and, in some cases, direction. Significant profile proximity was observed for cell adhesion molecules, adherens junction, axon guidance, spliceosome, glutamatergic synapse, serotonergic synapse, actin-cytoskeleton regulation, ferroptosis, synaptic vesicle cycle, and long-term potentiation.

The strongest proximity was observed for cell adhesion molecules, with Pearson's r = 0.549 and Spearman's rho = 0.399. Adherens junction had Pearson's r = 0.516 and Spearman's rho = 0.380. Axon guidance had Pearson's r = 0.371 and Spearman's rho = 0.312. Glutamatergic synapse had Pearson's r = 0.391 and Spearman's rho = 0.422, while serotonergic synapse had Pearson's r = 0.394 and Spearman's rho = 0.367. The synaptic vesicle cycle showed a more modest but significant proximity pattern, with Pearson's r = 0.323 and Spearman's rho = 0.302. Ferroptosis showed Pearson's r = 0.434 and Spearman's rho = 0.391. Actin regulation reached significance through Spearman correlation, with rho = 0.305 despite a lower Pearson correlation of 0.244.

These correlations indicate that the relative ordering of gene-level effects is partly shared. They do not show that the two phenotypes have the same overall pathway magnitude or the same direction of effect. Directional concordance was moderate, generally falling between 0.58 and 0.65. The synaptic vesicle cycle had a sign-concordance rate of 0.585, glutamatergic synapse 0.590, cell adhesion molecules 0.604, axon guidance 0.634, and adherens junction 0.646. The lowest concordance was observed for the renin-angiotensin system, with a rate of 0.467.

The distinction between proximity and concordance is important. Anorexia nervosa and binge-eating behaviour may involve the same molecular infrastructure while assigning different weights to particular genes, regions, or cellular states. This is compatible with a dimensional model in which synaptic, trafficking, structural, and metabolic components combine in different proportions. Such a model could eventually inform research stratification, but only after replication and validation against clinically meaningful outcomes.

The influential gene overlap supports the same interpretation. The analysis identified 154 genes influential in both diseases, 261 anorexia nervosa-specific influential genes, and 162 binge-eating-specific influential genes. The overlap was therefore substantial but incomplete. Shared genes included components of trafficking, actin remodeling, calcium signaling, phosphoinositide signaling, lysosomal biology, and synaptic function. Disease-specific genes included many candidates with larger influence in one phenotype or one tissue context.

Tissue and circuit heterogeneity adds important resolution

The tissue-level results provided a second form of divergence. In BE broad, the glutamatergic synapse was the most tissue-variable module, with a Z-score range of 4.10 between the amygdala, where the value was −2.95, and the anterior cingulate cortex BA24, where it was +1.15. GABAergic synapse showed a range of 3.59, from −2.55 in the amygdala to +1.04 in the anterior cingulate cortex. Neuroactive ligand-receptor interaction ranged from −1.27 in the amygdala to +1.85 in the caudate basal ganglia. The synaptic vesicle cycle reached its highest tissue-level value in the frontal cortex BA9 at +3.24, whereas its lowest tissue-level value was observed in the hippocampus at approximately +0.68. The spliceosome signal peaked in the nucleus accumbens basal ganglia at +3.20 and was lower in the caudate.

AN broad showed a different pattern. Axon guidance was strongest in the frontal cortex BA9 at +3.43 and weaker in the amygdala. The renin-angiotensin system varied from −2.92 in the nucleus accumbens to +0.85 in the anterior cingulate cortex. Ferroptosis ranged from −1.66 in the amygdala to +1.19 in the anterior cingulate cortex. RNA polymerase was most negative in the amygdala, with a Z-score of −2.39, whereas its frontal-cortex value was close to zero.

These results do not establish that one region causes one phenotype. They do show that meta-tissue aggregation can conceal region-specific contrasts. The pattern is compatible with broader neuroimaging work that distinguishes frontostriatal reward and reinforcement processes in binge-related phenotypes from the more distributed reward valuation, cognitive control, and interoceptive alterations described in anorexia nervosa [3-5].

The tissue results also reinforce the importance of cautious interpretation of meta-Z values. A positive meta-level statistic can reflect several moderate tissue contributions, a single strong tissue contribution, or a mixture of positive and negative regional effects. The same pathway may therefore have different functional meanings in the frontal cortex, amygdala, hippocampus, caudate, anterior cingulate, and nucleus accumbens.

Refined prioritization and experimental qualifications

The integrated prioritization identified three practical tiers. Tier 1 contained prioritized candidates RHOA, CACNA1B, SLC17A7, VAMP2, and ATP6V1E2. These candidates span trafficking and actin regulation, presynaptic calcium entry, vesicular glutamate loading, vesicle fusion, and vesicle acidification. Their value as candidates lies in the fact that they can be tested with functional assays rather than only expression measurements.

Tier 2 contained ARPC5L, ITGB1, SRC, PTK2, PIK3CA, PIK3CB, PAK2, PAK4, LIMK2, MYL9, VCL, and SSH2. These genes define the structural and signaling environment in which membrane trafficking and synaptic organization occur. They may be particularly useful for examining the relationship between RHOA activity, adhesion complexes, actin remodeling, and receptor recycling.

Tier 3 contained GNG12, GNG10, GNB2, ADCY3, MAP2K1, PLCB2, PLCB3, and PPP1CB. These genes showed substantial influence or strong disease divergence but may be more context-dependent. G-protein and phospholipase signaling genes were especially recurrent among the cross-module prioritized candidates. The complete 28-gene cross-module prioritized list included MAPK3, GNG12, MAPK1, RAF1, MAP2K1, GNG10, PLCB3, PLCB2, HRAS, GNB2, KRAS, GNG3, RHOA, PIK3CA, CAMK2B, ITGB1, MYL9, ADCY3, ITPR1, PIK3CB, SRC, PIK3R2, KCNJ3, ADCY6, GNG11, ITPR2, GNAQ, and ITPR3.

The 28 cross-module prioritized candidates are listed in Table 5.

**Table 5:** Core driver genes influential across at least three modules and both phenotypes Data analysis: leave-one-out (jackknife) influence analysis was used to identify genes recurring in at least three modules and both phenotypes; the reported percentages are influence summaries rather than independent effect estimates. These lists are prioritization short-lists only and do not imply causal disease genes.

Gene	Modules	Diseases	Mean absolute change (%)	Maximum change (%)	Sign flips
MAPK3	5	2	103.86	323.04	0
GNG12	5	2	103.56	351.58	2
MAPK1	5	2	87.61	260.58	0
RAF1	4	2	161.57	433.99	0
MAP2K1	4	2	130.38	331.95	2
GNG10	4	2	126.50	416.37	1
PLCB3	4	2	96.18	349.47	0
PLCB2	4	2	88.17	278.68	0
HRAS	4	2	79.06	188.06	0
GNB2	4	2	69.02	199.86	1
KRAS	4	2	60.43	152.98	0
GNG3	4	2	49.42	131.05	1
RHOA	3	2	165.81	535.92	2
PIK3CA	3	2	117.92	220.05	2
CAMK2B	3	2	115.75	242.28	0
ITGB1	3	2	97.85	250.27	0
MYL9	3	2	95.74	212.79	0
ADCY3	3	2	94.64	140.43	1
ITPR1	3	2	86.96	184.13	0
PIK3CB	3	2	83.44	161.32	2
SRC	3	2	83.44	266.70	1
PIK3R2	3	2	80.35	158.56	1
KCNJ3	3	2	62.42	129.58	0
ADCY6	3	2	59.87	109.96	0
GNG11	3	2	58.08	120.18	1
ITPR2	3	2	55.66	108.45	1
GNAQ	3	2	33.63	45.94	0
ITPR3	3	2	29.48	37.22	0

The leading robustness-weighted candidates are shown in Table 6.

**Table 6:** Top 15 genes in the Stage 3 robustness-weighted prioritization Data analysis: robustness-weighted prioritization combined module recurrence, disease breadth, maximum influence, and sign-flip status; this was a ranking procedure and did not generate independent p-values. The supplied composite score assigns positive weight to the presence of sign flips. Accordingly, it should be treated as a prioritization index rather than a pure stability measure.

Gene	Score	Modules	Diseases	Maximum influence (%)	Sign flips	Mean absolute Z
GNG12	0.933	5	2	351.58	2	2.2883
GNG10	0.894	4	2	416.37	1	3.3345
RHOA	0.850	3	2	535.92	2	7.1114
MAP2K1	0.847	4	2	331.95	2	2.5407
PPP1CB	0.775	2	2	866.02	2	6.8034
GNB2	0.774	4	2	199.86	1	3.5987
GNG5	0.742	4	1	367.89	1	2.6111
GNG3	0.736	4	2	131.05	1	1.6890
SRC	0.736	3	2	266.70	1	2.2640
MAPK3	0.717	5	2	323.04	0	2.2432
PIK3CA	0.710	3	2	220.05	2	1.6484
RAF1	0.704	4	2	433.99	0	2.4663
CACNA1B	0.703	4	1	296.48	1	2.2077
ARPC5L	0.700	1	2	491.79	2	4.0554
ITGB3	0.700	1	2	610.12	1	4.2548

These genes should not be interpreted as confirmed causal drivers. Leave-one-out influence identifies genes that contribute strongly to a summary statistic. It does not distinguish causal genes from correlated genes, pathway outliers, or genes with large Z-scores caused by LD or shared prediction architecture.

The Polycomb results provide a broader, hypothesis-generating context for biological systems that overlap with the NMN framework, especially synaptic vesicle dynamics, membrane trafficking, actin remodeling, lysosomal function, and cellular stress. They do not establish direct NMN sensitivity, NAD+ depletion, RAB11A involvement, or therapeutic efficacy of NMN. Neither RAB11A nor CPT2 appears among the top Polycomb-derived prioritized genes. The present results therefore supply a broader cellular context in which NMN-sensitive processes could be tested, but they do not constitute evidence that NMN or any NAD+-related intervention is therapeutically relevant to anorexia nervosa or binge-eating behaviour. Functional experiments must first establish disease-relevant cellular phenotypes before any NAD+ intervention is evaluated.

The appropriate translational question is therefore whether NAD+-related interventions alter functional synaptic, trafficking, or metabolic resilience under disease-relevant stress. The relevant outcome is not necessarily reversal of a specific TWAS Z-score. A treatment could improve vesicle recycling, mitochondrial reserve, or receptor trafficking without normalizing every genetically predicted expression association.

## Discussion

Conceptual advance

This study is a hypothesis-generating re-analysis of the same S-PrediXcan resource used in the companion work. It does not demonstrate altered neuronal function, pathway activation, or causal mechanisms.

**Figure 2:**
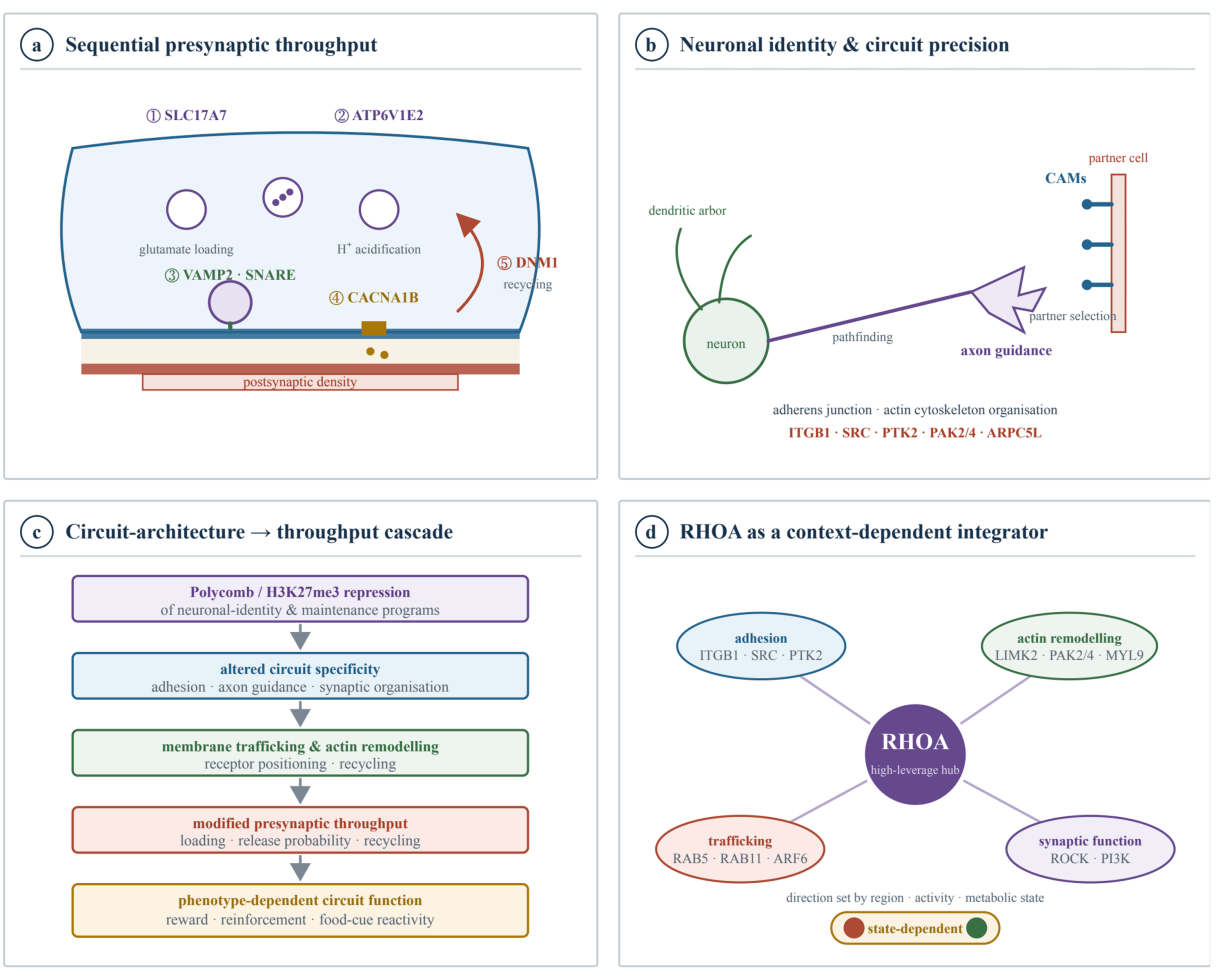
Mechanistic model of eating-disorder genetic risk within a Polycomb-regulated neuronal maintenance architecture (a) Genetic risk is proposed to converge on coordinated steps of presynaptic glutamatergic throughput: vesicular glutamate loading (SLC17A7/VGLUT1), lumenal acidification (ATP6V1E2), soluble N-ethylmaleimide-sensitive factor attachment protein receptor (SNARE)-dependent docking and fusion (VAMP2), voltage-gated calcium entry (CACNA1B), and recycling (DNM1). This motivates assays of quantal size, release probability, recycling kinetics, and synaptic fatigue under metabolic stress. (b) A neuronal identity and circuit-precision axis provides an upstream or parallel vulnerability, encompassing axonal pathfinding, dendritic self-avoidance, synaptic partner selection through cell-adhesion molecules (CAMs), and adherens-junction/actin-cytoskeleton organization (ITGB1, SRC, PTK2, PAK2/4, ARPC5L). Altered connectivity could reshape how presynaptic and neuromodulatory systems operate within networks. (c) Integrating both axes yields a hypothesis-generating "circuit-architecture to synaptic-throughput" model: Polycomb/H3K27me3-related programs derived from aging-neuron data are hypothesized to influence circuit specificity, with effects mediated through membrane trafficking and actin remodeling to modify presynaptic throughput. Relevance to reward processing, reinforcement learning, or food-cue reactivity requires testing. (d) RHOA is positioned as a high-leverage but context-dependent candidate network node bridging adhesion, actin remodeling, membrane trafficking, and synaptic function via RAB5/RAB11, ADP-ribosylation factor 6 (ARF6), ROCK, LIMK, and PI3K. Its effect direction may vary with brain region, neuronal activity, and metabolic state, making it a candidate for state-dependent experiments rather than simple directional validation. This figure depicts hypothesized mechanisms only; no analytical results are shown, and directionality is illustrative. The image was created by Ngo Cheung using Microsoft PowerPoint (Microsoft Corp., Redmond, WA, USA).

The central contribution of this analysis is the integration of three biological levels that were previously considered separately (Figure 2). The companion preprint emphasized synaptic vesicle dynamics, RHOA-centered trafficking, and mitochondrial substrate utilization. The current Polycomb results add neuronal identity, adhesion, circuit organization, and regional heterogeneity. Together, these findings are consistent with a hypothesis involving an aging-neuron-like vulnerability involving not one pathway but a network of cellular maintenance functions.

The strongest integrated result remains the strongest and most stable computational prioritization, the binge-eating-associated synaptic vesicle cycle. It is supported by a large meta-level Stouffer statistic, a competitive permutation p-value, a Wilcoxon test, a bootstrap interval excluding zero, tissue-level support, disease-differential testing, and survival of the pooled BH-FDR threshold. The result also has a mechanistic structure. SLC17A7, ATP6V1E2, VAMP2, CACNA1B, DNM1, and associated proteins map onto vesicle loading, acidification, calcium entry, fusion, and recycling.

The Polycomb analysis offers a hypothesis-generating structural context. Neuronal identity and adhesion programs may influence which synaptic contacts are formed and maintained. Axon guidance and adherens-junction signals may influence how circuits are organized. Actin and focal-adhesion pathways may regulate the positioning of receptors, channels, and vesicles. Presynaptic throughput may therefore be shaped by the architecture of the circuit in which it occurs.

This interpretation is compatible with the aging-neuron Polycomb model, but it should not be overstated. The current TWAS analysis does not show that H3K27me3 is altered in anorexia nervosa or binge-eating behaviour. It shows that eating-disorder genetic associations are enriched within gene programs derived from an aging-neuron chromatin analysis. The relationship is therefore one of conceptual convergence and hypothesis generation rather than independent mechanistic confirmation [12].

Implications for dimensional hypothesis generation

The moderate proximity and concordance results suggest that anorexia nervosa and binge-eating behaviour share molecular infrastructure more strongly than they share direction. This pattern provides a rationale for dimensional stratification. Instead of asking whether an individual has an "anorexia nervosa pathway" or a "binge-eating pathway," future studies could examine the relative contribution of several biological axes.

A synaptic-vesicle component could include SLC17A7, VAMP2, CACNA1B, ATP6V1E2, DNM1, and related genes. A trafficking and structural component could include RHOA, ARPC5L, ITGB1, SRC, PTK2, PAK2, PAK4, LIMK2, MYL9, and VCL. A metabolic-reserve component could include CPT2 and tricarboxylic acid (TCA)-cycle candidates, although these require stronger validation. A neuronal-identity component could include protocadherin and adhesion-related genes derived from the Polycomb program.

Such a framework would not be a diagnostic test. It would be a research model for relating molecular dimensions to clinical traits such as binge frequency, food-cue reactivity, cognitive restraint, impulsivity, interoceptive sensitivity, metabolic status, and treatment response. The value of this approach would depend on replication in independent samples and validation against clinically meaningful outcomes.

The model may also help generate hypotheses about why the same intervention could have different effects in different patients. A person whose dominant vulnerability lies in presynaptic fatigue may respond differently from someone whose dominant feature is impaired receptor recycling or altered metabolic reserve. RHOA may be especially useful as a state-dependent marker because its sign flips suggest that its functional effect may change with activity or metabolic context.

Translational roadmap

The first experimental stage should focus on presynaptic vesicle function. Neuronal or induced pluripotent stem cell (iPSC)-derived neuronal models can be used to examine SLC17A7-dependent vesicular glutamate loading, VAMP2-mediated docking and fusion, CACNA1B-dependent calcium entry, ATP6V1E2-related vesicle acidification, and DNM1-dependent recycling. Relevant measurements include miniature excitatory postsynaptic event amplitude, release probability, calcium currents, vesicle pH, vesicle recycling kinetics, and recovery after repeated stimulation.

Presynaptic calcium channels are particularly attractive because their abundance, localization, and coupling to release sensors influence the timing and strength of synaptic output [32]. The analysis should distinguish changes in channel number from changes in channel function or active-zone positioning. It should also test whether the relevant phenotype is altered basal transmission, abnormal facilitation, impaired replenishment, or accelerated fatigue.

Vesicle acidification should be evaluated together with recycling. vATPase activity creates the proton electrochemical gradient needed for neurotransmitter uptake, while clathrin uncoating controls when newly formed vesicles can acidify and refill [35]. This creates a direct functional link between ATP availability, membrane trafficking, and presynaptic transmission. NMN could be tested in this system, but only after baseline defects have been characterized.

The second stage should examine RHOA-dependent trafficking and actin remodeling. Experiments should measure RHOA-GTP, ROCK and LIMK activity, filamentous-actin organization, RAB5 and RAB11 compartment dynamics, endosomal pH, receptor internalization, and receptor recycling. Transferrin recycling and recycling of selected neurotransmitter or growth-factor receptors would provide tractable assays. These experiments should compare baseline and stress conditions, including repeated neuronal stimulation, nutrient deprivation, oxidative stress, and recovery after refeeding.

The third stage should examine neuronal adhesion and synaptic maintenance. ITGB1, SRC, PTK2, ARPC5L, PAK2, and PAK4 could be perturbed individually or in combination with RHOA. Readouts should include neurite morphology, dendritic spine density, synaptic puncta, adhesion-complex localization, actin organization, and the stability of synaptic contacts after repeated activity. This would test whether the adhesion and actin signals are merely statistical correlates or whether they influence synaptic resilience.

The fourth stage should examine metabolic reserve. CPT2 and TCA-cycle candidates should be tested with oxygen-consumption assays, spare respiratory capacity, mitochondrial membrane potential, NAD+/reduced NADH ratio, TCA-intermediate profiling, acylcarnitine measurements, and palmitate-supported respiration. A positive association with TCA genes should not be interpreted as evidence of healthy mitochondrial function. It may reflect increased demand, compensation, altered substrate availability, or inefficient oxidative metabolism.

Only after these functional phenotypes are established should NMN or related NAD+-augmenting interventions be assessed. The primary question should be whether such interventions improve functional resilience under stress. Relevant outcomes include recovery after repeated stimulation, vesicle recycling, receptor return to the cell surface, mitochondrial reserve, and resistance to oxidative or nutrient stress. The intervention should not be judged solely by whether it reverses a TWAS direction.

Limitations

First, the present work reuses the same underlying S-PrediXcan summary statistics as the companion study. It is therefore not an independent replication. Recurrence of a signal under a Polycomb-derived gene-set definition represents conceptual or hypothesis-generating convergence, not confirmation in an independent genetic resource.

Second, the analysis is based on genetically predicted expression rather than measured RNA, protein abundance, synaptic activity, vesicle release, mitochondrial flux, or chromatin occupancy. No colocalization or fine-mapping analysis was performed in the supplied workflow. S-PrediXcan findings can be affected by prediction-model quality, LD, correlated predictors, tissue mismatch, and horizontal pleiotropy.

Third, the multi-tissue Stouffer statistic assumes a degree of independence that may not hold for the six correlated brain tissues. The meta-analysis can improve ranking power but may overstate precision. A covariance-adjusted Brown or Kost analysis was not available in the supplied outputs. A positive meta-level value may represent a consistent multi-region pattern, a small number of dominant tissues, or a mixture of positive and negative regional effects. Tissue-specific analyses are therefore essential for interpretation.

Fourth, the supplied pipeline did not preserve the complete documentation of the upstream mouse-to-human orthology conversion. The orthology database and release, treatment of one-to-many mappings, and exact number of excluded genes were not available in the supplied execution record. These details must accompany the gene-set files and mapping table in the reproducibility deposit.

Fifth, many pathways overlap biologically and computationally. Cell adhesion, axon guidance, adherens junction, actin regulation, synaptic signaling, and neuroactive ligand-receptor pathways share genes and signaling components. The Stage 3 network captured this overlap through 24,225 Jaccard-defined edges, but the same network density can make pathway counts look more independent than they are. Gene-set redundancy should be considered in future analyses using overlap-aware clustering, competitive null models, or redundancy-reduction procedures [43].

Sixth, leave-one-out influence is not equivalent to causal importance. A gene may be influential because it has a large Z-score, because it is correlated with other genes, or because the gene set contains relatively few members. Sign flips are especially important because they indicate directional instability. The current robustness score gives positive weight to the presence of a sign flip, so it should be read as a prioritization heuristic rather than a pure measure of stability.

Seventh, even a fully documented mouse-to-human mapping depends on assumptions about cross-species conservation of gene function, regulatory organization, pathway membership, and neuronal biology. These assumptions may be particularly important for clustered protocadherins and other genes with species-specific organization [12,19].

Eighth, no prioritized gene or pathway was functionally validated in the present study. The mechanistic model, RHOA prioritization, and NMN-related hypotheses therefore require independent replication, cell-type-resolved analysis, and sequential experimental testing before biological or therapeutic claims can be advanced.

The competitive permutation p-value and Wilcoxon p-value answer different questions. A Wilcoxon result indicates that the genes in a set tend to shift away from zero. A competitive permutation result asks whether the set is more extreme than randomly selected genes. A pathway can pass one test but not the other. The axon-guidance and adherens-junction results illustrate this distinction: their Stouffer statistics were large, but their competitive p-values and bootstrap intervals were less secure than those of the binge-eating synaptic vesicle cycle.

Future directions

Future analyses should extend the current framework in four directions. First, the TWAS results should be followed by colocalization and fine-mapping for the highest-priority genes and pathways. This would help determine whether the gene-level associations and GWAS signals share causal variants or are linked through LD. Mendelian-randomization analyses may provide additional evidence, although they will require careful instrument selection and sensitivity testing [15,16].

Second, tissue resolution should be improved. Single-cell or cell-type-enriched TWAS models could determine whether the signals arise primarily in excitatory neurons, inhibitory neurons, glia, or mixed cell populations. Region-specific analyses could clarify why the frontal cortex, anterior cingulate, amygdala, caudate, hippocampus, and nucleus accumbens show different pathway patterns.

Third, functional models should incorporate disease-relevant genetic backgrounds. iPSC-derived neurons from individuals with anorexia nervosa- or binge-eating-associated genetic risk could be differentiated into excitatory and inhibitory lineages. Clustered regularly interspaced short palindromic repeats (CRISPR) interference, CRISPR activation, allele-specific perturbation, and rescue experiments could then test RHOA, ARPC5L, CACNA1B, SLC17A7, VAMP2, ATP6V1E2, DNM1, ITGB1, PTK2, and selected metabolic genes.

Finally, NMN experiments should be performed only after disease-relevant cellular phenotypes are established. The most informative design would compare baseline, repeated-activity, nutrient-stress, and oxidative-stress conditions, with and without NMN. Measurements should include NAD+/NADH balance, mitochondrial respiration, vesicle acidification, neurotransmitter release, receptor recycling, actin organization, and RHOA/ROCK activity. This approach would determine whether NAD+ augmentation changes functional resilience, rather than assuming that it should reverse a genetically predicted expression signal.

## Conclusions

The Polycomb-focused multi-gene-set TWAS prioritizes a coordinated neuronal-maintenance architecture-neuronal identity, adhesion, cytoskeletal remodeling, presynaptic vesicle cycling, and metabolic resilience, as a molecular dimension that cuts across anorexia nervosa and binge-eating behaviour. The strongest and most stable prioritization remains the binge-eating-associated synaptic vesicle cycle. All findings are computational associations derived from the same GWAS/TWAS resource used in the companion study; they require colocalization, cell-type-resolved models, and sequential functional assays, beginning with presynaptic and RHOA-dependent trafficking readouts, before any mechanistic or therapeutic claims can be advanced.

RHOA remains a high-priority candidate because of its recurrence across modules, high network degree, large influence magnitude, and repeated sign flips, but these features indicate prioritization and directional instability rather than a confirmed disease mechanism. The combined results are compatible with a dimensional model in which anorexia nervosa and binge-eating behaviour share molecular infrastructure but differ in pathway magnitude, regional weighting, and gene-level direction. NMN or other NAD+-related interventions should be evaluated only after disease-relevant cellular phenotypes are defined and under controlled stress conditions.
